# Potential use of *Bacillus paramycoides* for the production of the biopolymer polyhydroxybutyrate from leftover carob fruit agro-waste

**DOI:** 10.3934/microbiol.2022023

**Published:** 2022-08-08

**Authors:** Leila DJERRAB, Zohra CHEKROUD, Amer ROUABHIA, Mohamed Abdesselem DEMS, Imane ATTAILIA, Luis Isidoro Romero GARCIA, Mustapha Adnane SMADI

**Affiliations:** 1 Laboratory of Research on the Physical Chemistry of Surfaces and Interfaces (LRPCSI), University 20 Août 1955 Skikda, Algeria; 2 Research Laboratory of Interactions, Biodiversity, Ecosystems and Biotechnology (LRIBEB) University 20 Août 1955 Skikda, Algeria; 3 Research Laboratory of Biological Treatment of Residues, Department of Chemical Engineering and Food Technology, Institute of Vitivinicultural and Agri-food Research (IVAGRO), University of Cadiz, Spain; 4 Bioinformatics and Biostatistics Unit (BIBSU), National Center for Biotechnology, Constantine, Algeria; 5 Superior School of Food Sciences and Agro Food Industries (ESSAIA), Algiers, Algeria; 6 Director Laboratory of Biological Treatment of Residues, Department of Chemical Engineering and Food Technology University of Cadiz, Spain; 7 Laboratory of Animal Biotechnologies, National Center for Biotechnology, Constantine, Algeria

**Keywords:** Bacillus paramycoides, carob waste, crotonic acid, polyhydroxybutyrate (PHB), valorization

## Abstract

This study was designed to investigate, at a laboratory scale, the possibility of valorizing the leftover carob fruits to produce the eco-friendly biopolymer polyhydroxybutyrate (PHB) by using the bacterial strain *Bacillus paramycoides*, which has been isolated from the botanical garden of Skikda University in Algeria. The PHB production was tested under various conditions: a pH of 3–8, temperature range of 30–44 °C, carob extracted molasses concentration of 2–8% v/v, an incubation time of 24–96 h and an agitation speed of 150–300 rpm. The effects of different nitrogen sources and carob extracted molasses treatment types were also investigated. The PHB concentration was determined quantitatively as crotonic acid by measuring the absorbance at 300 nm. Cell growth was quantified by measuring the density of the culture at 600 nm. The presence of PHB was confirmed by applying high-performance liquid chromatography (HPLC) using an Aminex HPX-87H and implementing gas chromatography analysis. The best yield of PHB synthesis was obtained by using 6% v/v of 5 M H_2_SO_4_ treated with carob molasses as a carbon source, with peptone as a nitrogen source; incubation was conducted at 37 °C for 96 h at an agitation speed of 300 rpm (114.95 mg/L). The HPLC analysis confirmed the synthesis of PHB by *B. paramycoides* to have a chromatogram retention time of 22.5 min. Carob waste was successfully valorized to PHB.

## 1. Introduction

Carob (*Ceratonia siliqua*) is a wildly cultivated tree in the Mediterranean countries. It belongs to the legume group and has a high capacity to grow on water-poor land [1]. In Algeria, this plant is frequently cultivated. In 2011, it was estimated that the carob cultivation occupied an area of 1000 ha, where 69.58% of the cultivated area was in the Bejaia region (north-central Algeria); additionally, [2] states that the annual Algerian production of carob reached 4042 tons in 2019. Although carob is known worldwide for its therapeutic effects or in the agro-food industry as an ingredient in human and animal food, it is still poorly exploited in Algeria. Crops are usually left in the trees unharvested to be eaten by animals or fallen, remaining on the ground as agricultural waste.

Developing an eco-friendly chemical industry has become an urgent necessity, where both industry and research direct their efforts toward exploiting agricultural biomass sources [3]. Carob fruit is very rich in sugars. Many researchers have studied the possibility of carob waste revalorization for biofuel production (e.g., ethanol) [1],[4] or biohydrogen production [5]. Cheap, natural agro-waste products like carob leftovers have also been used as feedstock alternative [6] sources for producing polyhydroxyalkanoates (PHAs). The accumulation of plastic materials originating from petrochemicals has become a serious environmental problem due to their versatile qualities of lightness, durability and resistance to degradation. Thus, researchers have been investigating the production of bioplastic materials, an environmentally friendly alternative to petrochemical plastics [7] that is biologically degradable by microbial enzymes into CO_2_, methane, H_2_O and biomass [8]. They also have important properties like biocompatibility and non-toxic performance [9].

PHAs are a class of polyesters that Lemoigne, a French chemist, discovered in 1926 in *Bacillus megaterium* [10]; they can be synthesized by using a variety of bacteria, such as *Staphylococcus*, *Alcaligenes*, *Pseudomonas*, *Micrococcus*, *Rhodococcus* and *Cupriavidus necator*, as intracellular compounds and energy-storage materials when nutritional elements, such as nitrogen, phosphorus, sulfur, oxygen and magnesium, are limited and the provided carbon source is in excess [11],[12].

This study examines the possibility of valorizing carob fruit remains at a laboratory scale. We applied a safe biological technique by transforming agricultural waste into the ecological biopolymer polyhydroxybutyrate (PHB) (a group of PHAs) using a bacterial strain of *Bacillus paramycoides*. The latter was isolated from the botanical garden of the University of Skikda in Algeria. It was identified using the 16S rRNA sequencing method. To the best of our knowledge, this is the first time that this bacterial species has been confirmed to produce PHB from carob waste.

## 2. Materials and methods

### 2.1. Collection and preparation of leftover carob fruits

In October 2020, leftover Carob fruits were collected from private farms in Collo, which is in the northeast of Algeria. The agricultural waste was immediately sorted and washed in hot distilled water to remove dirt and other debris; it was then cut into small pieces (2 cm × 2 cm) and dried in an oven for 30 min at 40 °C. The production of PHB via bacteria requires large amounts of sugars. Carob fruit waste was first transformed into molasses. The molasses extraction was done according to the method reported by Dhaouadi and his collaborators [13], with some modifications. Carob fruit that had been cut into small pieces (2 cm × 2 cm) was dissolved overnight to obtain a syrup. The obtained syrup was boiled and concentrated to a thick, dark syrup. It was then sterilized via tyndallization in order to eliminate the microbial charge without causing thermal degradation of the sugars. The final molasses was stored in sterilized dark bottles at room temperature.

### 2.2. Physico-chemical characterization of molasses from carob fruit waste

#### 2.2.1. Centrifugation

The untreated molasses solution was prepared in a 250-mL beaker. A total of 15 g of molasses were weighed and diluted in 100 mL of distilled water. The clarifying procedure was made by centrifuging the molasses solution for 15 min at 3500 rpm and filtering it using Whatman No. 1 filter paper. The resulting solution was then employed for further investigation [14].

#### 2.2.2. Hydrolytic pretreatment of molasses

The solution was hydrolyzed by adding 5 mL of 5 M or 1.5 M sulfuric acid or 3 M HCl to 100 ml of the solution and incubating it at 90 °C in a water bath for 1 h. The pH was kept at 3.0 using 1 M NaOH. After incubation and cooling, the molasses solutions were centrifuged again at 8000 g for 20 min. The supernatants were adjusted to pH 7.0 [15]. The yield of hydrolysis was calculated using the following formula [16]:

$$H\%\frac{Ar0-Ar}{1.5027S0\left(At0-Ar0\right)}$$ (1)

where *Ar* and *Ar*0 denote the total reducing sugar (TRS), a measurement of glucose and fructose concentrations, after and before acid hydrolysis, respectively, *S*0 is the approximate sucrose, 1.0527 is the stoichiometric correction factor and *At*0 is the total sugar content before acid hydrolysis.

#### 2.2.3. Physico-chemical analysis

The soluble chemical oxygen demand (COD), pH, total solids (TSs) and volatile solids (VSs) were determined according to standard methods AWWA-WPCF [17]. The pH was measured daily using a pH meter (Crison Basic 20). The TSs were weighted after drying the molasses sample at 110 ± 5 °C. For VS determination, the dried samples were calcined at 550 ± 5 °C. The alkalinity was determined directly by applying acid titration to the raw samples. A lixiviation process (10 g of sample in 100 mL of deionized water for 20 min) was performed, followed by filtration through 0.47-µm and 0.22-µm glass fiber filters, to analyze the COD, which was measured by performing spectrophotometric absorbance measurements using a spectrophotometer. The TRS in the crude molasses content was measured before and after molasses hydrolysis by using the 3,5-dinitrosalicylic acid method [18]. The sugar concentration was confirmed by applying high-performance liquid chromatography (HPLC) provided with a refractive index (R1) detector (50 °C) and a column (SH1011, 8.0 × 300 nm, Shodex) [19].

### 2.3. Screening of a bacterial strain producing PHB

The producing PHB bacterial strain was isolated from a soil sample collected from the botanic garden of Skikda University by performing serial dilutions. Pure, viable colonies were then grown in a mineral salt medium (MSM) [20] to which 2% glucose and 10 g of agar were added; the mixture was then incubated for 48 h at 37 °C. The PHB-producing colonies were detected by adding an ethanolic solution of 3% Sudan Black B (C29H24N6 224-087, Sigma). The Sudan Black B positive isolate was tested for its ability to produce PHB in the agar MSM medium [20] and added 2% of carob molasses instead of glucose. The bacterial strain was spotted onto the MSM agar medium and incubated at 37 °C for 48 h. The bacterial colonies were colored with an ethanolic solution of 3% Sudan Black B (C29H24N6 224-087, Sigma) to confirm its ability to produce PHB [21].

### 2.4. Bacterial strain identification

The strain subjected to PHB production testing using carob molasses was identified based on its 16S rRNA sequencing results. The extraction of the bacterial genomic DNA was performed using the GF-1 Nucleic Acid Extraction Kit according to the manufacturer's instructions. Polymerase Chain Reaction (PCR) amplification was achieved using the primer set of 16S rRNA genes (27F: 5′–AGAGTTTGATCCTGGCTCAG–3′ and 1492R 5′–CCGTCAATTCCTTTGAGTTT–3′) [22]. PCR products were separated using a 1.5% agarose gel electrophoresis. The bacterial strain was identified by comparing consensus sequences to a database library of known 16S rRNA gene sequences in GenBank under the conditions of multiple sequence alignment (http://wwwncbi.nlm.nih.gov/blast/Blast.cgi).

### 2.5. Optimization of physico-chemical PHB production conditions

An inoculum from the chosen bacterial strain was prepared by inoculating a loop of the bacterial culture into 100 mL of nutrient broth in 250 mL conical flasks.

The inoculated flasks were incubated for 24 h on a rotary shaker (150 rpm) at 37 °C. Cells were then collected by performing centrifugation at 10,000 g for 15 min at 4 °C before being washed aseptically with sterile distilled water and resuspended in 250-mL Erlenmeyer flasks containing 100 ml of mineral salt medium (MSM) [20]. Then, 2% (v/v) centrifuged carob molasses was added. The flasks were incubated in a rotary incubator for 24, 48, 72, or 96 h at agitation rates of 150, 200 or 300 rpm. To test the effects of physico-chemical factors on PHB production and cell growth, the pH was adjusted to 7.0, 3.0 or 8.0 by manually adding NaOH (2 M) or HCl (2 M) and applying a temperature of 30, 37, or 44 °C. Ammonium chloride in the MSM medium (2.3 g/L) was replaced by applying an equivalent concentration of yeast extract, peptone, beef extract and ammonium sulfate one at a time. After determining the best physico-chemical conditions, the centrifuged carob molasses was replaced with crude and hydrolyzed molasses by adding 5 M or 1.5 M sulfuric acid or 3 M HCl. In the last stage, sulfuric acid (5 M) pretreated molasses concentration was elevated from 2% (v/v) to 4, 6 or 8% (v/v).

### 2.6. Spectrophotometric analysis

The quantity of accumulated PHB was measured by performing spectrophotometric analysis. Bacterial cells containing the polymer were collected after 10 min of centrifugation at 4000 rpm. The pellets were resuspended in an equivalent amount of 4% sodium hypochlorite and incubated for 1 h at 37 °C. The pellet was washed with acetone, ethanol and distilled water to eliminate any remaining undesirable elements. The mixture was centrifuged once again, and the supernatant was discarded. The cells containing the polymer granules were dissolved in boiling chloroform to extract the PHB, which was then allowed to evaporate [23]. A quantity of 10 mL of concentrated sulfuric acid (98%) was added to the residue. The tubes were kept in a boiling water bath for 15 min. The solution was left to cool. The PHB was determined quantitatively as crotonic acid by measuring the absorbance at 300 nm using an ultraviolet (UV) spectrophotometer with H_2_SO_4_ solution as a blank. The amount of PHB in the bacterial cells was determined in milligram per liter by comparing the absorbance readings to a standard crotonic acid curve [23].

### 2.7. Cell growth

Cell growth, an indicator of carob molasses consumption, was monitored by measuring the optical density of the cultures at 600 nm using a UV spectrophotometer (Rs 232). Ten milliliters of the culture medium were centrifuged at 10,000 rpm and 4 °C for 15 min, and the cell pellets were washed with 10 mL of distilled water. The cell pellets were then dried at 105 °C for 48 h, or until a constant weight was obtained. The standard calibration curve was used to determine the cell mass concentration at OD600 [24] after diluting the sample to appropriate concentrations. The cell dry weight (CDW) was determined in mg/L.

### 2.8. HPLC analysis

Samples ranging from 0.01 to 500 mg of PHB-containing material were digested in 1 mL of concentrated sulfuric acid at 90 °C for 30 min. The tubes were cooled on ice, and 4 mL of 0.014 N H_2_SO_4_ were added under rapid mixing. Before HPLC analysis, the samples were diluted 5 to 100 times with 0.014 N H_2_SO_4_ containing 0.8 mg of adipic acid (Sigma) per mL as an internal standard; they were then filtered through a membrane filter with a pore size of 0.45 µm (HAWP, Millipore Corp) to remove particulate material. The injection volumes ranged from 10 to 50 µL and the sample concentrations from 0.2 to 560 µg/mL. Samples were eluted with 0.014 N H_2_SO_4_ at a 0.7 mL/min flow rate from an Aminex HPX-87H ion exclusion organic acid analysis column (C18 4.6 × 250) (Torrance, CA, USA) preceded by an Aminex HPX-85X ion exclusion guard column. HPLC analysis was performed using either a Waters Associates 6000A solvent delivery system with a U6K injector, or a series of three chromatographs (PerkinElmer, Inc., Norwalk, Conn.) with a variable loop injector. The absorbance of crotonic acid was measured at 214 nm (Waters 441 absorbance spectrophotometer) or 210 nm spectrophotometer. The amount of crotonic acid produced from PHB was calculated by using the regression equation derived from known crotonic acid standards [25].

### 2.9. Gas chromatography analysis for PHB determination

The PHB extracted using chloroform and sulfuric acid was analyzed by applying gas chromatography (GC) (Shimadzu^®^, GC-2014). Each sample was subjected to methanolysis before the GC analysis. It was prepared according to the method reported by Braunegg et al. [26]. The GC analysis was characterized by a Chrompack CPSIL-5CB column (25 m × 0.25 mm × 0.25 µm), a flame ionization detector set to 250 °C and nitrogen as the carrier gas. Each 1-mL sample was washed with 0.2 mL of sodium hypochlorite, and the mixture was centrifuged at 10000 rpm for 10 min. The organic phase was discarded and dissolved in 2 mL of methanol solution (3% v/v H_2_SO_4_) and 1 mL of chloroform. The tubes were incubated at 100 °C for 4 h. After cooling, 1 mL of distilled water was added and the tubes were agitated to separate the phases. Finally, 1 µL of the collected organic part was injected into the GC system. A calibration curve was obtained via the injection of poly(3-hydroxybutyric acid-co-3-hydroxyvaleric acid) with a poly(3-hydroxyvalerate) (PHV) content of 8%, which was obtained from Sigma–Aldrich, Milan, Italy. It was submitted to the same procedure applied to the biomass samples. Heptadecane was applied as an internal standard at a concentration of 0.1 g/L [27].

### 2.10. Statistical analysis

All tests were conducted in triplicate. The data were analyzed by performing a one-way analysis of variance (ANOVA), followed by pairwise comparisons via the Fisher's least significant difference (LSD) post hoc test, which allowed us to examine the difference between the effects of different conditions. Statistical significance was considered at P < 0.05. Data processing was done using Statistica 10 software (StatSoft, Inc.).

## 3. Results and discussion

### 3.1. Bacterial production of PHB from carob molasses

**Figure 1.**
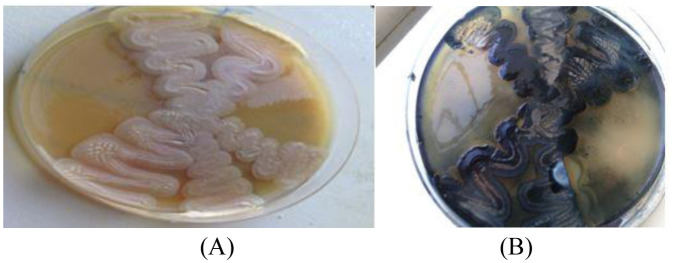
Colonies of the bacterial strain that produced PHB from carob molasses. (A) Before Sudan Black B staining; (B) after Sudan Black B staining.

A wide variety of Gram-positive and Gram-negative bacteria are known to accumulate PHA utilizing cheap nutrient as a source of carbon [9]. In the present study, a PHB-accumulating bacterial strain was successfully isolated from the botanical garden of the University 20 Août 1955 in Skikda, Algeria. The change of the colonies' color to dark greenish-blue after the addition of Sudan Black B indicates that it is PHB-producing strain [7]. Different bacterial species, such as *Bacillus subtilis*, *Bacillus licheniformis*, *Bacillus cereus*, *Bacillus megaterium* and *Bacillus thuringiensis* were screened based on the Sudan Black B staining test [28],[29]. The bacterial strain was then tested for its ability to produce PHB using carob molasses instead of glucose in the MSM medium. The isolate revealed positive Sudan Black B test results (Figure 1), which confirmed the production of PHB using the cheap agro-waste carob molasses. The carob molasses was characterized by a high content of total sugars, i.e., 116.86 g/L, among which 94.35 g/L was sucrose (Table 1).

**Table 1.** Physico-chemical characteristics of crude carob molasses.

Samples	Parameters								Types of sugars		
Molasses of carob	TS (g/L)	TS (%)	VS (g/L)	VS (%)	COD (g/L)	pH	Alkalinity (g/L)	Total sugars (g/L)	Sucrose	Glucose	Fructose
	797.5	79%	796.95	79.69%	106	5.61	20.6	116.86	94.35	15.407	7.108

Soluble chemical oxygen demand (COD), Total solids (TSs), Volatile solids (VSs), Total reducing sugars (TRSs).

### 3.2. Bacterial strain identification

The chosen bacterial strain was identified by performing 16S rRNA gene sequencing and comparing consensus sequences to a database library of known 16S rRNA gene sequences from GenBan (http://www.ncbi.nlm.nih.gov/blast/Blast.cgi). Sequence alignment, which was performed using BLASTN software to compare up to 1,500 bp, attributed a high identity (99.43%) to the Bacillus paramycoides strain MCCC 1A04098, which was subjected to partial 16 rRNA sequencing National Center for Biotechnology Information (NCBI) assessing the number NR_157734.1 (Table 2).

**Table 2.** *Bacillus paramycoides* strain MCCC 1A04098 16S rRNA partial sequencing results.

Description	Scientific Name	Max Score	Total Score	Query Cover	E Value	Per Ident	Acc Len	Accession
*Bacillus paramycoides strain MCCC 1A04098 16S* rRNA partial sequence	*Bacillus paramycoides*	1905	1905	100%	0.0	99.43	1509	NR157734.1

E Value: Expect value, Per Ident: percent Identity, ACC Len: Accesson length.

### 3.3. Optimization of physico-chemical culture conditions

#### 3.3.1. Effects of different incubation periods on bacterial growth and PHB production

**Figure 2.**
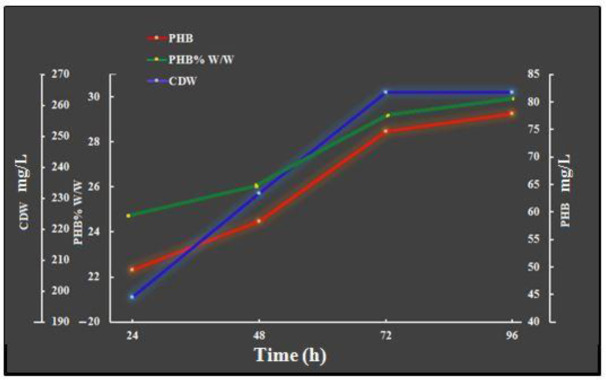
Effects of different incubation periods on bacterial growth and PHB production under the conditions of 37 °C, a pH of 7, 150 rpm with shake flasks and a batch culture.

Optimization of the cultivation parameters and recovery processes are among the strategies now used to improve PHB production [30]. Figure 2 shows the effects of time on cell growth and PHB production. The results indicate that, at 96 h of incubation, the maximum CDW and PHB accumulation were obtained (264.24 mg/L and 77.837 mg/L, respectively) with a yield of 29.45% PHB per cell dry weight. This demonstrates that the biomass and PHB production were consistent with the growth circumstances, and that a strain's PHB production is connected to its biomass. As biomass production increases, bacteria tend to collect PHB to reach maximum levels [31]. Our results are in agreement with those obtained by the authors of [30], who used sugar cane molasses as a carbon source for the production of PHB via *Bacillus subtilis* and *Escherichia coli* [32]. Sen et al. [33] revealed that 72 h is the best incubation period for polymer production via various carbon sources. Vijay and Tarika [34] reported that 24 h is the best time for PHB production via *Staphylococcus aureus*. The authors of [35],[36] recorded a duration of 48 h.

#### 3.3.2. Effects of agitation rate on bacterial growth and PHB production

**Figure 3.**
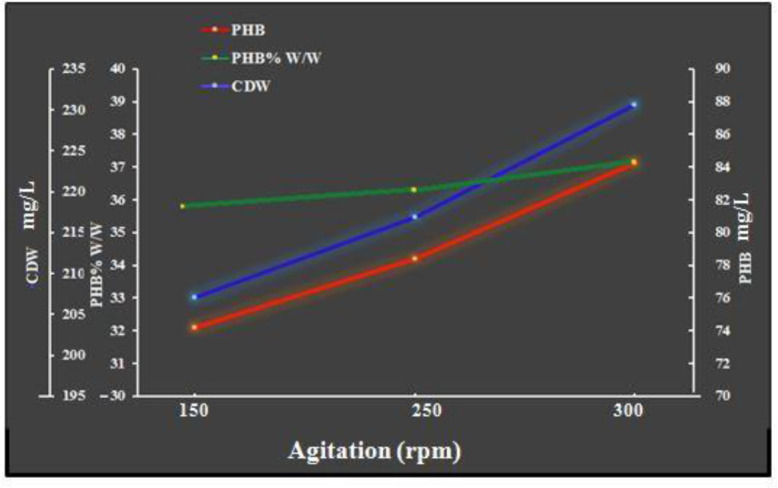
Effects of agitation rate on bacterial growth and PHB production after 96 h of incubation under the conditions of 37 °C, a pH of 7, the use of shake flasks and the implementation of a batch culture.

Agitation is crucial for a homogeneous cell, mixing, mass and heat dispersion [37]. It also promotes aeration for the cells by increasing the oxygen transfer rate throughout the fermentation medium [21]. Figure 3 indicates that 300 rpm is the best agitation rate for the production of PHB and the growth of *Bacillus paramycoides* (84.24 mg/L and 230.62 mg/L, respectively) with a yield of 36.52% PHB per cell dry weight. Increasing the agitation rate appeared to increase cell proliferation. A slower agitation speed may result in cell aggregation, making the culture media more diverse. Moreover, it can decrease cell proliferation and reduce PHB production [21]. Our results are partially in agreement with [39], where the maximum PHB production via *Bacillus* sp. 1 was obtained at 300 rpm. In contrast, the optimum PHB accumulation via *Bacillus* sp. 2 was obtained at 200 rpm. The optimum speed reported by the authors of [15] for PHB production via *Cupriavidus necator* was 250 rpm. The authors of [38] found that 200 rpm was the optimum speed for achieving highly efficient PHB fermentation via *Bacillus flexus*.

#### 3.3.3. Effects of pH on bacterial growth and PHB production

**Figure 4.**
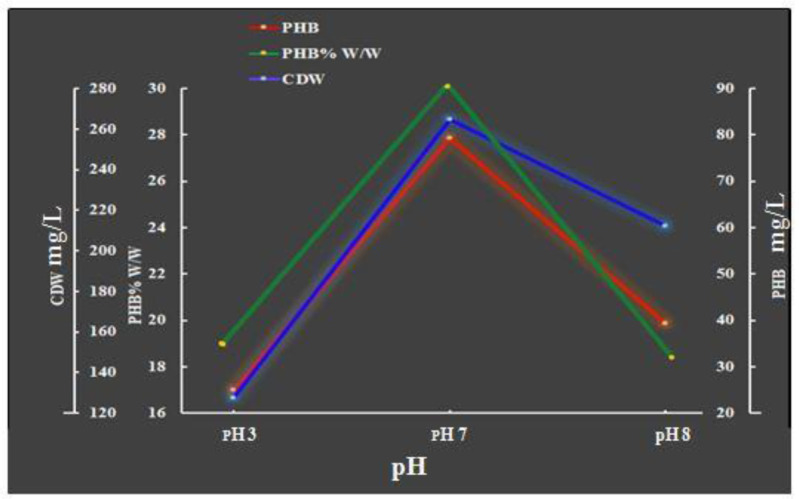
Effects of pH on bacterial growth and PHB production after 96 h of incubation under the conditions of 37 °C and 300 rpm with shake flasks and a batch culture.

The influence of the pH of the medium on cell growth and PHB production revealed that a pH of 7 was the best pH to achieve the maximum PHB accumulation and maximum *Bacillus paramycoides* biomass growth (79.34 and 264.67 mg/L, respectively), with a yield of 29.97% PHB per cell dry weight (Figure 4). The same results were recorded by the author of [32]. The bacilli may grow at a medium pH ranging from 3 to 9, with an optimum pH of 7 [38].

#### 3.3.4. Effects of temperature on cell growth and PHB production

**Figure 5.**
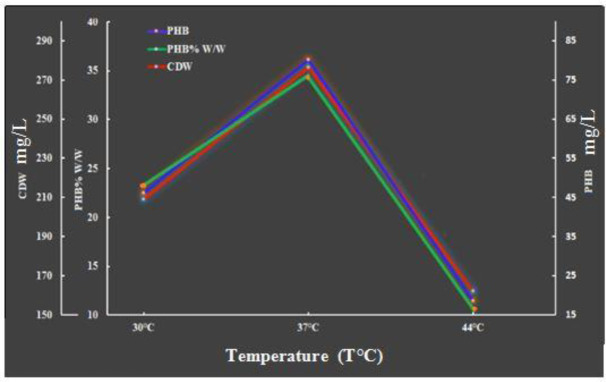
Effect of temperature on cell growth and PHB production after 96 h of incubation under the conditions of a pH of 7 and 300 rpm with shake flasks and a batch culture.

Figure 5 shows the influence of culture temperature on cell development and PHB generation. The temperature effect was studied between 30 and 44 °C. The results showed that the maximum CDW and PHB biopolymer concentrations were obtained at a culture incubation temperature of 37 °C (276.33 and 80.167 mg/L, respectively), with a yield of 29.011% PHB per cell dry weight. Similar results were reported in [40] and [36]. Other studies have found that some *Bacillus* species require an incubation temperature of 30 °C to achieve the highest quantity of PHB [34].

### 3.4. Effects of various nitrogen sources on bacterial growth and PHB production

**Figure 6.**
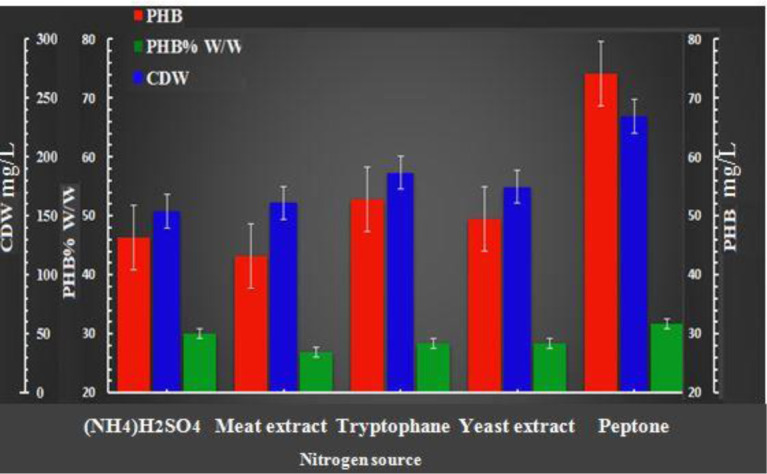
Effects of various nitrogen sources on bacterial growth and PHB production after 96 h of incubation under the conditions of 37 °C, a pH of 7 and 300 rpm with shake flasks and a batch culture. The data represent the mean of three different readings (± standard deviation).

Under restricted growth conditions, bacterial cells synthesize PHB polymer when the carbon source is rich and nitrogen, phosphorus, magnesium, sulfur or oxygen is present in limiting concentrations. The nitrogen source is one of the factors affecting cell growth and PHB production [37]. In this study, the effects of different nitrogen sources were investigated. Figure 6 indicates that peptone gave the maximum growth and PHB production (234.33 mg/L and 74.09 mg/L, respectively) with a yield of 31.61% PHB per cell dry weight, followed by tryptophan (186.67 µg/mL and 52.813 mg/L, respectively) with 28.29% PHB per cell dry weight and yeast extract (174.67 and 49.503 mg/L, respectively) with 28.34% PHB per cell dry weight. A decrease in the cells' biomass and PHB content was observed when we used meat extract (154 mg/L and 46.29 mg/L, respectively), which yielded 30.058% PHB per cell dry weight. and ammonium sulfate (NH_4_)_2_SO_4_ (61 µg/mL and 43.174 µg/mL, respectively), which yielded 26.81% PHB per cell dry weight. This observation indicates that peptone's and tryptophan's amino acids and peptides may increase PHB accumulation [40].

### 3.5. Optimization of carob molasses properties

#### 3.5.1. Effects of untreated and pretreated carob molasses on cell growth and PHB production

**Figure 7.**
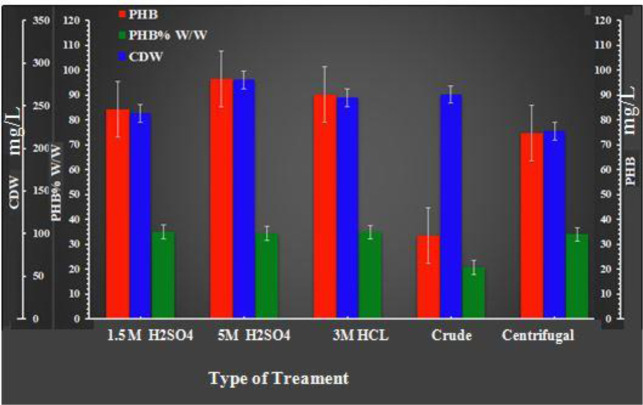
Effects of untreated and pretreated carob molasses after 96 h of incubation under the conditions of 37 °C, a pH of 7 and 300 rpm with shake flasks and a batch culture. The data represent the mean of three different readings (±standard deviation).

Figure 7 shows that the higher PHB contents were obtained with hydrothermal acid-pretreated molasses compared to crude molasses. When using acid-hydrolyzed molasses, the PHB yield ranged between 34.98% and 34.45%. The highest cell biomass and PHB contents were recorded when the molasses was hydrolyzed with 5 M H_2_SO_4_ (280 mg/L and 96.46 mg/L, respectively), followed by the molasses treated with 3 M HCl, which yielded a 259.33 mg/L CDW and 90.33 mg/L of PHB. In the case of the molasses hydrolyzed with 1.5 M H_2_SO_4_, we obtained a 241 mg/L CDW and 84.31 mg/L of PHB.

The lowest contents of PHB were produced by *Bacillus paramycoides* when crude molasses was used (20.58 mg/mL), despite the large cell biomass (CDW: 263 mg/L); additionally, the PHB yield production was only 7.83%, whereas centrifuged molasses (CDW: 220 and PHB content: 74.64 mg/L) yielded 33.92% PHB per dry weight. Our results agree with those reported in [32], which states that acid-hydrolyzed molasses increased PHB accumulation relative to that yielded by untreated molasses. This can be explained by the low concentrations of simple sugars (glucose and fructose) in the untreated molasses [41]. Effectively, carob molasses contains high levels of sucrose (94.35 g/L) and low levels of simple sugars like glucose (15.407 g/L) and fructose (7.108 g/L).

The rate of sucrose hydrolysis and invert sugar production increased directly with increasing sulfuric acid concentration employed in the molasses pretreatment [34]. It reached 69.38% with 5 M H_2_SO_4_, 63.59% with 1.5 M H_2_SO_4_ and 61.32% with 3M HCl (Table 3). The increase in PHB content was, however, weakly proportional with the yield of carob molasses hydrolysis. In fact, with a hydrolysis yield of 69.38%, the PHB content increased from 74.64 mg/L in the case of the centrifuged molasses, to 96.46 mg/L in the case of the 5 M H_2_SO_4_ hydrolyzed molasses. This may be explained by the fact that glucose uptake by *Bacillus paramycoides* is not completely metabolized by the cells due to the accumulation of toxic by-products such as hydroxymethylfurfural [33], in addition to the use of a part of glucose for cell biosynthesis.

**Table 3.** Molasses hydrolysis conditions and the yield obtained.

Method	Temperature (°C)	Time (min)	pH	Yield (%)
Acid 3 M (HCl)	90	60	3	63.59
Acid 5 M (H_2_SO_4_)	90	60	3	69.38
Acid 1.5 M (H_2_SO_4_)	90	60	3	61.32

#### 3.5.2. Effects of acid-pretreated carob molasses concentration on cell growth and PHB production

**Figure 8.**
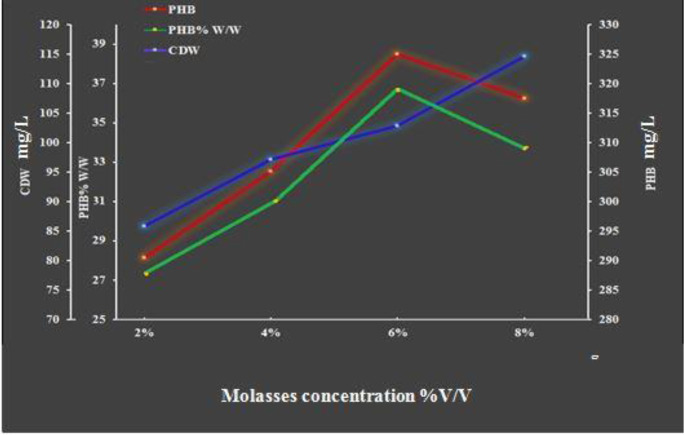
Effects of acid pretreated carob molasses concentration after 96 h of incubation under the conditions of 37 °C, a pH of 7 and 300 rpm with shake flasks and a batch culture.

To investigate the influence of acid-pretreated carob molasses concentration on PHB production and CDW, the initial concentration of carob molasses pretreated with 5M H_2_SO_4_ was elevated from 2% to 8%. Figure 8 shows that the PHB content and CDW increased with the increase of carob molasses concentration. The highest PHB yield was obtained at 6% molasses (36.52% PHB per cell dry weight), where the PHB content reached its maximum (114.9 mg/L). A slight decrease in PHB content was observed at 8% molasses (107.5 mg/L, 33.10% PHB per cell dry weight), with an increase in the cell biomass (324.7 mg/L). This can be explained by the fact that *Bacillus paramycoides* started degrading the accumulated PHB by using depolymerase enzymes [42] for cell growth. Similar observations were reported in [32]. The main drawback of the overall PHB production costs is the high cost of the carbon substrates selected as feedstocks. An economical and frugal carbon substrate minimizes the final product's viable total market cost [43].

### 3.6. Statistical analysis

Table 4 lists the ANOVA results. A very large significant difference between the different physico-chemical conditions can be noted. For PHB content, P = 0.000, and 0.000 < P < 0.003 for CDW. The results of multiple comparisons of the means, which were achieved via the LSD post hoc test at P < 0.05, indicate that the most cell growth and largest PHB amount could be achieved by using 6% v/v of 5 M hydrolyzed molasses as a carbon source and peptone as a nitrogen source, and incubating the samples at 37 °C for 96 h at an agitation speed of 300 rpm.

### 3.7. HPLC-based identification of PHB

**Figure 9.**
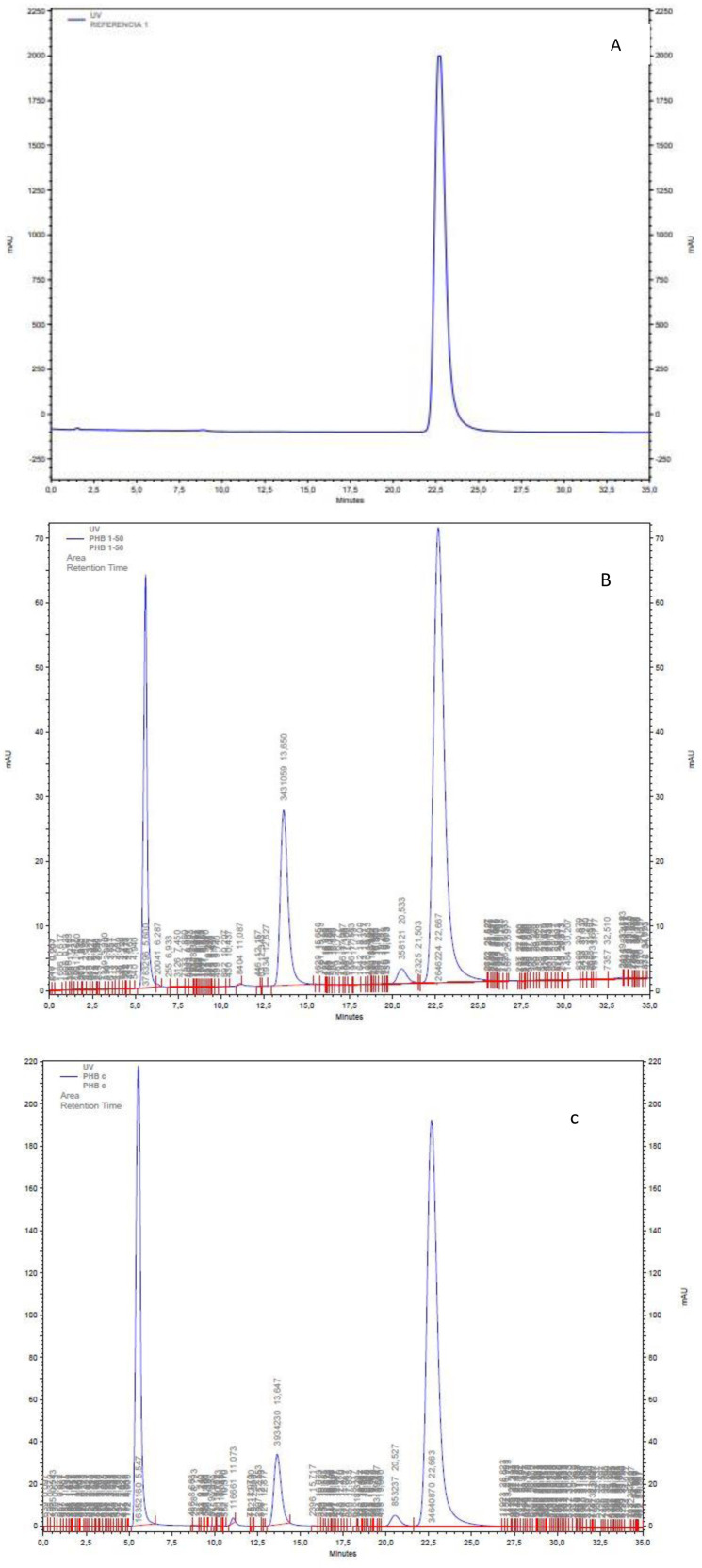
HPLC analysis of crotonic acid. (A) Crotonic acid at 22.5 min; (B) HPLC analysis of pure PHB digested directly in concentrated sulphuric acid. (C) PHB produced via fermentation using carob molasses as a carbon source treated in concentrated H_2_SO_4_. The chromatographic conditions are given in the text. The 6- and 12.5-min peaks represent the solvent front and adipic acid internal standard, respectively.

The production of PHB via *Bacillus paramycoides* was confirmed using the HPLC technique [25],[44], which depends on the conversion of the extracted PHB with concentrated sulfuric acid to crotonic acid [25]. Crotonic acid may be measured in samples containing 0.01 to 14 µg of PHB [45]. Recrystallized crotonic acid in 0.014 N H_2_SO_4_ revealed a single peak with a retention time of 22.5 min (Figure 9A). Figure 9B shows the analysis results for pure PHB following sulfuric acid conversion to crotonic acid, while Figure 9C shows that for PHB as a result of using *Bacillus paramycoides* treated directly with sulfuric acid.

A comparison of the three chromatograms revealed that they have the same main peak and the same crotonic acid retention time (22.5 min), which confirms the presence of PHB in the samples extracted from *Bacillus paramycoides*.

**Table 4.** Experimental ANOVA results.

	*P*		Post Hoc Test	
Experimental conditions	CDW	PHB	CDW	PHB
T (°C)	0.000	0.000	T-37^(1)^, T -44^(1)^, T -30^(2)^	T -37^(1)^, T -44^(2)^, T°C-30^(3)^
pH	0.000	0.000	7^(1)^, 8^(2)^, 3^(3)^	7^(1)^, 8^(2)^, 3^(3)^
Time (h)	0.000	0.000	T_96_^(1)^, T_72_^(1)^, T_48_^(2)^, T_24_^(3)^	T_96_^(1)^, T_72_^(2)^, T_48_^(3)^, T_24_^(4)^
Nitrogen source	0.000	0.000	N_Peptone_^(1)^, N_Tryptophan_^(2)^, N_Yeast extract_^(3)^, N_Meat extract_^(4)^ N_NH4SO4_^(5)^	N_Peptone_^(1)^, N _Tryptophan_^(2)^, N_Yeast extract_^(3)^, N_Meat extract_^(4)^, N_NH4SO4_^(5)^
Agitation (rpm)	0.003	0.000	Ag_300_^(1)^, Ag_200_^(2)^, Ag_150_^(2)^	Ag_300_^(1)^, Ag_200_^(2)^, Ag_150_^(3)^
Untreated and pretreated carob molasses	0.000	0.000	Hy_5M H2SO4_^(1)^, Hy_3MHCl_^(3)^, Hy_1.5M H2SO4_^(4)^, _Centri_^(5)^, _Crude_^(2)^	Hy_5M H2SO4_^(1)^, Hy_3MHCl_^(2)^, Hy_1.5M H2SO4_^(3)^, _Centri_^(4)^, _Crude_^(5)^_,_
Carob molasses concentration (%)	0.000	0.000	Mc_8%_^(1)^, Mc_6%_^(1)^, Mc_4%_^(2)^_,_ Mc_2%_^(3)^	Mc_8%_^(1)^, Mc_6%_^(1)^, Mc_4%_^(2)^_,_ Mc_2%_^(3)^

**CDW**: Cell dry matter; **PHB**: polyhydroxybutyrate; different letters in brackets indicate significant differences according to the ANOVA test results followed, by the post-hoc Fisher test (LSD) with a 95% confidence interval; **T**: temperature, **N**: nitrogen source; **Ag**: agitation rate; **Hy**: hydrolyzed carob molasses; **Centri**: centrifuged molasses; **Crude**: crude molasses; **Mc**: Molasses concentration.

### 3.8. GC analysis of the extracted biopolymer

**Figure 10.**
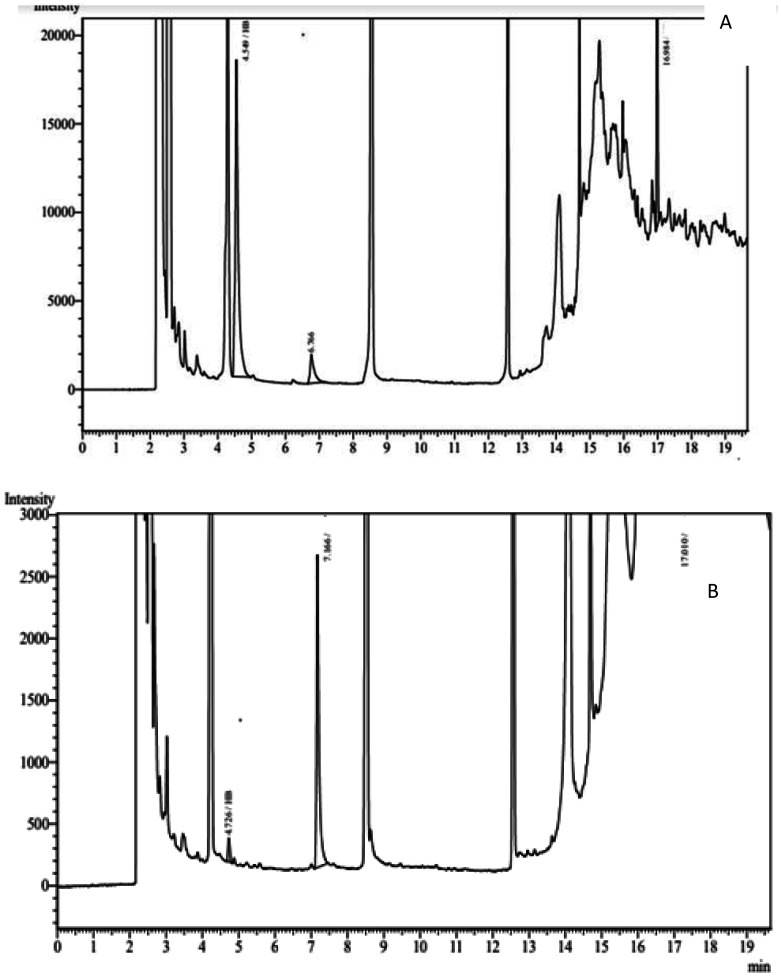
GC analysis of *Bacillus paramycoides* synthesized biopolymer. (A) Chromatograms of the stander poly(3-hydroxybutyric acid-co-3-hydroxyvaleric acid), (B) chromatograms of the biopolymer synthesized by *Bacillus paramycoides*.

Comparing the chromatograms of standard poly(3-hydroxybutyric acid-*co*-3-hydroxyvaleric acid) (Figure 10A) with the chromatograms of the biopolymer extracted from *Bacillus paramycoides* revealed that the bacteria-derived biopolymer contained two major types of polymers, i.e., PHB and PHV, corresponding to retention times of 4.447 and 6.599 min, respectively (Figure 10B).

## 4. Conclusions

This study investigated the valorization of the carob fruit. We applied a safe biological technique by transforming agricultural waste using a bacterial strain of *Bacillus paramycoides*. The results indicate that the Algerian agro-waste, i.e., leftover carob fruit, can be considered an eco-friendly PHB biopolymer feedstock. The production of PHB was confirmed via HPLC analysis with an Aminex HPX-87H system and GC analysis. The highest PHB content was obtained after 96 h of incubation at 37 °C and a pH of 7, as well as 6% of 5 M H_2_SO_4_-pretreated carob molasses. The PHB physico-chemical characteristics were under study to confirm the possibility of producing PHB at an industrial scale and commercializing it to produce biodegradable bioplastic, which will help to solve the problem of non-degradable petroleum plastic.
